# Complete genome sequences of *Rhizobium* sp. strain SL42 and *Hydrogenophaga* sp. strain SL48, microsymbionts of *Amphicarpaea bracteata*

**DOI:** 10.3389/frmbi.2024.1309947

**Published:** 2024-02-13

**Authors:** Gayathri Ilangumaran, Sowmyalakshmi Subramanian, Donald Lawrence Smith

**Affiliations:** Department of Plant Science, McGill University, Sainte-Anne-de-Bellevue, QC, Canada

**Keywords:** whole genome sequencing, Illumina, nanopore, gene functions, biosynthetic gene clusters

## Abstract

This study comprehensively analyzed two distinct rhizobacterial strains, *Rhizobium* sp. SL42 and *Hydrogenophaga* sp. SL48, through whole genome *de novo* sequencing. Isolated from root nodules of *Amphicarpaea bracteata*, a native legume related to soybean, they were selected to explore beneficial rhizobacteria from native plant relatives. Utilizing Illumina and Nanopore sequencers and MaSuRCA assembly, their complete genetic information was elucidated. *Rhizobium* sp. SL42 has a 4.06 Mbp circular chromosome and two plasmids with 60% GC content, while *Hydrogenophaga* sp. SL48 exhibits a 5.43 Mbp circular chromosome with 65% GC content. Genetic analysis identified them as new species, supported by ANI values (77.72% for SL42 and 83.39% for SL48) below the threshold. The genomic analysis unraveled a plethora of genes encoding diverse metabolic functions, secretion systems for substance transport, quorum sensing for coordination, and biosynthetic gene clusters suggesting the production of bioactive compounds. These functional properties contribute to plant growth stimulation, reflecting the symbiotic relationship of rhizobacteria with plants, potentially involving nitrogen fixation and growth-promoting compounds. This research contributes valuable knowledge about plant-microbe interactions and plant growth promotion by these two strains of rhizobacteria.

## 1 Introduction

Plants belonging to the Leguminosae family engage in symbiotic relationships with nitrogen-fixing bacteria, commonly referred to as rhizobia, which reside within their root nodules. These nodules are known not only to harbor nitrogen-fixing symbionts but also other bacteria that potentially function as plant growth-promoting rhizobacteria (PGPR) (Bai et al., 2002). *Amphicarpaea bracteata* (L.) Fernald, a wild legume native to North America, particularly Canada and the lower 48 states of the USA, is one such plant of interest. It predominantly thrives in woody, shaded areas of wetlands but can also be found in similar non-wetland habitats in some regions. This herbaceous perennial species, which grows into a vine, annually produces flowers, pods, and seeds (USDA, 2017). Its seeds and roots are edible, and indigenous communities have historically used it for both nutritional and medicinal purposes (Moerman, 1998).

Importantly, *A. bracteata* shares a close evolutionary relationship with the cultivated soybean (*Glycine max* (L.) Merill) and hosts symbiotic bacteria from the *Bradyrhizobium* genus, albeit with different genotypes (Sterner and Parker, 1999). In a prior study, we isolated culturable members of the *A. bracteata* nodule phytomicrobiome and examined their potential benefits for soybean plants (Ilangumaran et al., 2021). Two isolates, *Rhizobium* sp. SL42 and *Hydrogenophaga* sp. SL48, emerged as promising candidates by enhancing salt tolerance and promoting soybean growth and maturity under controlled conditions (Ilangumaran et al., 2021, 2022). The taxonomy, morphological and physiological characteristics of these two strains are given in **Table 1**. Members of the Genus *Rhizobium* are well-known for their ability to form nodules and fix atmospheric nitrogen, underscoring the significance of the *Rhizobium* sp. SL42 isolate. Additionally, *Rhizobium* sp. SL42 was also capable of forming small nodules with soybean plants (data not shown). The isolation of a *Rhizobium* species from *A. bracteata* nodules is an intriguing discovery since the dominant symbiont is *Bradyrhizobium* (Sterner and Parker, 1999). Furthermore, the genus *Hydrogenophaga* is composed of bacteria that utilize hydrogen as an energy source, oxidizing it through the enzyme hydrogenase (Contzen et al., 2000). Our previous studies (cited above) have revealed associations between *Hydrogenophaga* species and plant roots, a novel finding. However, the molecular functions related to their roles in promoting plant growth and protection remain unexplored.

**Table 1:** Taxonomic classification and general features of *Rhizobium* sp. SL42 and *Hydrogenophaga* sp. SL48.

Property	*Rhizobium* sp. SL42	*Hydrogenophaga* sp. SL48
Classification: Domain	Bacteria	Bacteria
Phylum	Proteobacteria	Proteobacteria
Class	Alphaproteobacteria	Betaproteobacteria
Order	Rhizobiales	Burkholderiales
Family	Rhizobiaceae	Comamonadaceae
Genus	*Rhizobium*	*Hydrogenophaga*
Species	unidentified	unidentified
Gram stain	Negative	Negative
Cell shape	Rod	Rod
Motility	Motile	Motile
Temperature range	Mesophile	Mesophile
Optimum temperature	25-30 °C (min. temp. 4 °C)	25-30 °C (min. temp. 4 °C)
pH range; Optimum	7.0	7.0
Carbon source	Mannitol	Mannitol
Habitat	Soil, root nodule on host	Soil, root nodule on host
Salinity	Up to 250 mM NaCl	Up to 100 mM NaCl
Oxygen requirement	Aerobic	Aerobic
Biotic relationship	Free-living/symbiont	Free-living/symbiont
Pathogenicity	Non-pathogenic	Non-pathogenic
Biosafety level/Risk Group	1	1
Isolation	Root nodule of *Amphicarpaea bracteata*	Root nodule of *Amphicarpaea bracteata*
Geographic location	Sainte-Anne-de-Bellevue, Canada	Sainte-Anne-de-Bellevue, Canada
Latitude	45.404 °N	45.404 °N
Longitude	73.934 °W	73.934 °W
Altitude	50 m	50 m
Sample collection	July 2017	July 2017

The primary objective of this study is to sequence the genomes of the isolates *Rhizobium* sp. SL42 and *Hydrogenophaga* sp. SL48 using high-throughput next-generation sequencing technology. We aim to comprehensively analyze the whole genome sequences using available platforms to characterize the features of the genome that are relevant to the plant growth-promoting characteristics of these bacteria.

The significance of whole genome sequencing of beneficial rhizobacteria are substantial for advancing our understanding of microbial-mediated plant growth promotion. This research elucidates the genomic underpinnings that enable these bacteria to enhance plant development, offering tangible prospects for agricultural innovation. By mapping the genomes of these microorganisms, we gain insights into the natural mechanisms that can be harnessed to amplify crop health and yield. This knowledge not only advances our fundamental understanding of symbiotic relationships in agriculture but also holds promise for the development of regenerative approaches. Recognizing these genetic attributes could lead to the adoption of rhizobacteria in agricultural systems, contributing to a more sustainable and robust agroecosystems. Such biotechnological applications of beneficial rhizobacteria stand to revolutionize agricultural practices, paving the way towards an environmentally friendly and resilient agricultural future.

## 2 Materials and methods

### 2.1 Bacterial culture and whole genome sequencing

The isolates *Rhizobium* sp. SL42 and *Hydrogenophaga* sp. SL48 were initially grown on yeast extract mannitol (YEM) agar plates at 25°C from glycerol stock cultures. Single well-developed colonies were cultivated in YEM broth and incubated at 25°C, 150 rpm, until they reached an OD_600nm_ of 1.0. Following incubation, the cells were collected by centrifugation at 5000 × g for 10 minutes and then resuspended in 5 mL of broth.

Genomic DNA extraction was performed from 2 mL of cells using the DNeasy Ultraclean Microbial Kit (QIAGEN, Venlo, Netherlands). The isolated DNA, with a concentration of 15 μg in 25 μl Tris buffer, was subsequently dried using a vacuum-free evaporator (Centrifan PE, KD Scientific, Holliston, USA) and stored in DNAStable (Biomatrica, San Diego, USA) for *de novo* whole genome sequencing at Genotypic Technology Ltd., Bangalore, India.

The samples underwent quality control, showing optimal yield and concentration (**Supplementary Table S1**). DNA integrity was evaluated by agarose gel electrophoresis (**Supplementary Figure S1**). For quality control and Sanger sequencing, the concentration and purity of the genomic DNA were assessed using the Nanodrop Spectrophotometer 2000 (Thermo Fisher Scientific, Waltham, USA) and the Qubit dsDNA HS assay kit (Thermo Fisher Scientific, Waltham, USA). PCR amplification of *16S rRNA* was conducted using 30-50 ng of the genomic DNA as the template with Takara ExTaq (Takara Bio, Shiga, Japan) and the resulted PCR product was purified and subjected to Sanger sequencing (Sanger et al., 1977) (**Supplementary Table S1**).

For Illumina sequencing, libraries were constructed using the Nextera XT DNA Library Preparation protocol (Illumina, San Diego, USA). Illumina-compatible sequencing libraries were quantified using the Qubit fluorometer and their fragment size distribution analyzed on Agilent TapeStation. The libraries were then sequenced on an Illumina HiSeq X Ten sequencer (Illumina) using 150 bp paired-end chemistry. In preparation for Nanopore sequencing, the Native barcoding kit (EXP-NBD114) from Oxford Nanopore Technology (ONT, Oxford, GB) was used. Native barcode ligation was performed, followed by adapter ligation. The sequencing library was eluted for sequencing on GridION X5 (ONT) using SpotON flow cell R9.4 (FLO-MIN106) in a 48-hour sequencing protocol. Nanopore raw reads (in ‘fast5’ format) were base-called (in ‘fastq5’ format) (Wick et al., 2019) using Fast base calling configuration and de-multiplexed using Guppy v2.3.4.

Quality control, trimming, and initial analyses were conducted using Commander, an NGS analysis tool developed by Genotypic Technology, Bangalore, India. Trimgalore, a standard tool (https://www.bioinformatics.babraham.ac.uk/projects/trim_galore/), was utilized to remove low-quality reads from Illumina raw data, while Nanopore raw reads underwent processing with Porechop (https://github.com/rrwick/Porechop). Only high-quality reads were retained from both platforms. The quality threshold value for Illumina data filtering was Q30 and Nanopore data filtering was Q7.

Hybrid assembly was performed using MaSuRCA v3.3.7 hybrid assembler (Zimin et al., 2013). Gene prediction was carried out using PROKKA (Seemann, 2014), and the predicted proteins were annotated against the UniProt protein database using the DIAMOND BLASTp program for gene ontology and annotation (Buchfink et al., 2015). Pathway analysis utilized the Kyoto Encyclopedia of Genes and Genomes (KEGG) orthology (KO) database of molecular functions (Kanehisa et al., 2016) to assign KO identifiers to genes and proteins and generate pathway maps. The assembled genome was also used for Simple Sequence Repeats (SSR) prediction using the MISA (MIcro SAtellite identification tool) software (Beier et al., 2017) (**Supplementary Figure S2**). Additionally, the genome was independently annotated by the NCBI Prokaryotic Genome Annotation Pipeline (PGAP) (Tatusova et al., 2016).

### 2.2 Genome analysis

#### 2.2.1 Phylogenetic analysis using IQ-TREE

To analyze the phylogenetic relationships of two bacterial strains based on their 16S rRNA sequences, we first retrieved related sequences through NCBI’s BLAST search (Zhang et al., 2000). Highly similar sequences were optimized for Megablast (Morgulis et al., 2008), with an expectation threshold of 0.05, and a target of 100 sequences, deliberately excluding those from uncultured clones. The sequences were then aligned using Multiple Sequence Comparison by Log-Expectation – MUSCLE (Edgar, 2004) integrated into EBI sequence analysis tools (Madeira et al., 2022), resulting in a multiple sequence alignment (MSA) file in Phylip interleaved format. Subsequently, IQ-TREE (Nguyen et al., 2014) was employed for constructing maximum likelihood phylogenetic trees using its standard settings. The number of bootstrap alignments and maximum iterations were set to 1000, with a correlation coefficient of 0.99, to ensure robust tree construction. It conducted both the Ultrafast Bootstrap analysis (Hoang et al., 2017) and the SH-aLRT branch test (Guindon et al., 2010). The input MSA files for *Rhizobium* had 22 sequences with 1360 nucleotide sites, and *Hydrogenophaga* had 20 sequences with 1409 nucleotide sites. IQ-TREE included ModelFinder (Kalyaanamoorthy et al., 2017) analysis, which identified the HKY+F+I model as the best fit according to Bayesian Information Criterion (BIC). The resulting maximum-likelihood trees were generated in Newick format. For visualization and interpretation, iTOL v5 (Letunić and Bork, 2021) was used, where the trees were displayed and annotated, rooted at their outgroup taxon, and bootstrap values were presented. Statistical measures such as Log-likelihood, AIC, and BIC scores, as well as the identification of near-zero internal branches, provided insights into the robustness and reliability of the phylogenetic trees.

#### 2.2.2 Genome comparison visualization

The circular representation of the chromosome for *Rhizobium* sp. SL42 and *Hydrogenophaga* sp. SL48 was generated using CGView, a tool that facilitates visualizing genomic structures and the arrangement of open reading frames (ORFs). Additionally, the Blast ring image generator (BRIG) circular map was employed to facilitate a detailed comparison between the genomes of *Rhizobium* sp. SL42 and *Hydrogenophaga* sp. SL48 with those of other closely related species in the *Rhizobium* and *Hydrogenophaga* genera available in the NCBI Genome database (Alikhan et al., 2011).

#### 2.2.3 Genome data-mining

The genomes of *Rhizobium* and *Hydrogenophaga* were compared with those in the NCBI database using the M1CR0B1AL1Z3R web server – https://microbializer.tau.ac.il/ (Avram et al., 2019). This server uses algorithms like Prodigal for ORF extraction, MMSEQS2 for homolog detection, and MCL for clustering. Orthologous groups were analyzed using MAFFT for amino acid sequence alignment and RAxML for species tree inference based on core-proteome alignment. The pan-genome analysis included GC content, ORF number distribution, and orthologous group size distribution. Only complete, fully assembled genomes were used to accurately identify orthologs and study horizontal gene transfer and bacterial evolution.

#### 2.2.4 Identifying biosynthetic gene clusters

To search the genome sequences for secondary metabolite biosynthetic gene clusters, *fasta* files of *Rhizobium* sp. SL42 and *Hydrogenophaga* sp. SL48 genome sequence were queried for antiSMASH 5.0 bacterial sequence analysis. The genomes were analyzed using KnownClusterBlast, ClusterBlast, SubClusterBlast, MIBiG cluster comparison, ActiveSiteFinder, RREFinder, Cluster 5 Pfam analysis, Pfam-based GO term annotation and TIGRFam analysis to identify as many gene clusters as possible (Blin et al., 2019).

## 3 Results

### 3.1 Genome properties

The samples underwent quality control, showing optimal yield and concentration (**Supplementary Table S2**). DNA integrity was evaluated by agarose gel electrophoresis (**Supplementary Figure S2**). Based on the *16S rRNA* gene sequence analysis, strain SL42 was identified as *Rhizobium* with 98% identity to *Rhizobium ipomoeae* strain NFB1 and is closely related to the taxon *R. ipomoeae* shin9-1T (TaxID: 1210932) and the type strain was isolated from a water convolvulus field (Sheu et al., 2016). Strain SL48 was identified as *Hydrogenophaga* with 99% identity to *Hydrogenophaga taeniospiralis* CCUG 15921T strain NBRC 102512 and it was the most closely related type strain (TaxID: 1281780). *De novo* sequencing hits for SL42 were for *Rhizobium* with 98% identity and SL48 for *Hydrogenophaga* with 93% identity. Further analysis was performed using reference genomes available on NCBI, *Rhizobium flava* YW14 (TaxID: 1335061) for SL42 and *Hydrogenaphaga pseudoflava* DSM 1034 (TaxID: 47421) for SL48. The average nucleotide identity – ANI (Yoon et al., 2017) values were compared with the reference genomes of the type strains of closely related species, and both strains exhibited values (77.72% for SL42 and 83.39% for SL48) well below the threshold level for designation as novel species.

The Illumina-compatible sequencing library had an average fragment size of 580 bp (**Supplementary Figure S3**), and pre-processing retained over 2 million paired-end reads for both samples (**Supplementary Tables S3–S12**). MaSuRCA v3.3.7 was used for hybrid assembly of Illumina and Nanopore reads, with sufficient sequencing coverage. The genome of *Rhizobium* sp. SL42 consists of a 4.06 Mbp circular chromosome and two circular plasmids of 750237 bp and 351829 bp with a GC content of 60%. The genome of *Hydrogenophaga* sp. SL48 consists of one 5.43 Mbp circular chromosome with a GC content of 65% (**Table 2**). Whole genome sequences were submitted to NCBI genome database (**Table 3**).

**Table 2:** Assembly statistics for whole genome sequencing and annotation summary of *Rhizobium* sp. SL42 and *Hydrogenophaga* sp. SL48.

Assembly statistics	*Rhizobium* sp. SL42	*Hydrogenophaga* sp. SL48
Contigs Generated	3	1
Contig Chromosome 1	4063937 bp	5433040
Contig Plasmid 1	351829 bp	
Contig Plasmid 2	750237 bp	
Total Contigs Length	5166003	5433040
N50 value	4063937	5433040
GC mol%	60	65
Coverage Illumina	166.96	181.35
Coverage Nanopore	127.73	115.18
Annotation	*Rhizobium*	*Hydrogenophaga*
CDS (genes)	4727	5077
Proteins (Annotated)	4642	4937

**Table 3:** Whole genome sequencing project information NCBI Genome database.

	*Rhizobium* sp. SL42	*Hydrogenophaga* sp. SL48
Name	*Rhizobium* sp. strain:SL42 Genome	*Hydrogenophaga* sp. strain:SL48 Genome
Accession number	CP063397; CP063398; CP063399	CP063400
BioProject	PRJNA669345	PRJNA669344
BioSample	SAMN16451206	SAMN16451201
Locus Tag	IM739	IM738
Tax ID	1210932	1904254
Genome size	4.06 Mbp	5.43 Mbp
Assembly method	MaSuRCA 3.3.7	MaSuRCA 3.3.7
Assembly name	MGM_Rhim_1	MGM _Hyga_1
Reference Title	Genome sequence of *Rhizobium* sp. strain SL42	Genome sequence of *Hydrogenophaga* sp. strain SL48
Reference authors:	Ilangumaran, G., Subramanian, S., and Smith, D.	Ilangumaran, G., Subramanian, S., and Smith, D.

### 3.2 Insights from the genome sequencing

Gene prediction and annotation were conducted for both *Rhizobium* sp. SL42 and *Hydrogenophaga* sp. SL48, revealing comprehensive gene ontology and protein predictions (**Supplementary Figures S4, S5**). Our genomic analysis uncovered a diverse array of predicted genes associated with various biological functions. These gene clusters encompassed flagella, chemotaxis, homoserine lactone, and multidrug resistance, along with regulatory and transport proteins. Notably, *Rhizobium* sp. SL42 possessed genes encoding Type I and Type IV secretion systems, while *Hydrogenophaga* sp. SL48 carried genes for Type II and Type IV secretion systems, as well as hydrogenase enzymes. Both strains also exhibited genes related to essential cellular processes, including photosystem I, nodulation, nitrogen fixation, heat shock, cold shock proteins, hypoxic response, iron chelation, and carotenoid synthesis. Genomic features contributing to these biological functions and related plant-microbe interactions is presented in **Table 4**.

**Table 4:** Genes related to PGPR attributes in the genome of *Rhizobium* sp. SL42 and *Hydrogenophaga* sp. SL48.

*Rhizobium* sp. SL42		
Gene	Function	# Genes encoding
bcr	Bicyclomycin resistance	3
bdlA; bigR	Biofilm	6
cspA; cspE; cspG	cold shock protein, cold shock-like protein	7
entS; fepC; fepD; fepG	Enterobactin	7
hspQ	heat shock protein	1
rhtB	homoserine lactone efflux protein	7
hrp1	hypoxic response protein	1
yfeA; hemH	Iron chelation	2
lptA; lptB; lptG; lapA; lapB	lipopolysaccharide assembly and export proteins	8
mdtA; mdtB; mdtC; mdtN; mdtK; mdtE	Multidrug resistance	24
mrpA; mrpB; mrpC; mrpD; mrpE; mrpG; mrpF	Na(+)/H(+) antiporter subunit	7
fixK	nitrogen fixation regulation	4
nodM, nolR	Nodulation	1
pleC	Non-motile and phage-resistance protein	3
envZ	Osmolarity sensor protein	1
hemF	Oxygen-dependent coproporphyrinogen-III oxidase	1
ycf3; regA	Photosynthesis	2
crtI; crtB; carA2	Phytoene	3
	Putative signaling	22
fpvA; fhuA; ftsY; chvE, cheD; fhuE;	Receptor	12
aroK; aroA; aroE; quiA	Shikimate pathway	5
chaA	Sodium-potassium/proton antiporter	1
potA; potB; potD	Spermidine/putrescine	25
gerE	Spore germination protein	1
soj	Sporulation initiation inhibitor	2
prsD; prsE;	Type I secretion system	15
virB4; virB9; virb10, virB11	Type IV secretion system	4
clcB	Voltage-gated ClC-type chloride channel	1
*Hydrogenophaga* sp. SL48		
	Acid shock protein	1
arpC	Antibiotic efflux pump outer membrane protein	1
bcr	Bicyclomycin resistance	2
icaR	Biofilm operon regulator	1
ble	Bleomycin resistance	1
kfoC	Chondroitin synthase	2
cspA; cspG	cold shock protein, cold shock-like protein	2
fas6	Cytokinin riboside 5’-monophosphate phosphoribohydrolase	1
entS	Enterobactin	1
fbpC	Fe(3+) ions import ATP-binding protein	1
hslR	heat shock protein	1
rhtB	homoserine lactone efflux protein	5
hypF; hypB; hypD	Hydrogenase maturation factor	3
hrp1	hypoxic response protein	1
hemH; sirB	Iron chelation	2
lptA; lptB; lptC; lptG; lptF; lapA; lapB	lipopolysaccharide assembly and export proteins	16
mdtB; mdtN; mdtE mdtA; mdtC; mexR; mexA; mdtD; mdtH; mdtG	Multidrug resistance	13
gerN; mrpA; mrpD; mrpE; mrpG; mrpF; mnhC1	Na(+)/H(+) antiporter subunit	7
hoxF; hoxU; hoxY; hoxH	NAD-reducing hydrogenase HoxS subunit	4
fixK	nitrogen fixation regulation	1
nifH; nifD; nifK	Nitrogenase iron protein	5
nifW	Nitrogenase-stabilizing/protective protein	1
nodD	Nodulation protein	4
envZ	Osmolarity sensor protein	2
osmY	Osmotically-inducible protein Y	3
hemF	Oxygen-dependent coproporphyrinogen-III oxidase	1
	Periplasmic [NiFeSe] hydrogenase subunit	2
kcsA	pH-gated potassium channel	1
regA	Photosynthesis	1
crtB	Phytoene	1
	Putative signaling	1
cheD; cirA; aer; ftsY; fhuA; chvE; fucA; fhuE;	Receptor	8
cbbS1; cbbL; rlp2	Ribulose bisphosphate carboxylase	3
rubA; hrb	Rubredoxin	2
aroL; aroA; aroE; quiA; ydiB	Shikimate pathway	6
chaA	Sodium-potassium/proton antiporter	1
potA; potB; potD;	Spermidine/putrescine	12
spsA	Spore coat polysaccharide biosynthesis protein	1
srkA	stress response kinase A	1
iaaM	Tryptophan 2-monooxygenase	1
xpsD; gspE; gspF; epsE; epsF; hxcR; xcpQ; xcpV; xcpT; pulD;	Type II secretion system	13
virB1; virB4; virB8; virb10, virB1; ptlf	Type IV secretion system	7

The KEGG pathway mapping further elucidated the functions associated with the predicted proteins in these bacteria, encompassing bacterial motility proteins, secretion system proteins, bacterial chemotaxis, flagellar assembly, peptidoglycan biosynthesis, and quorum sensing. Intriguingly, each strain possessed unique proteins that set them apart. In *Rhizobium* sp. SL42, proteins related to photosynthesis, carbon fixation, and carotenoid biosynthesis pathways were identified, while in *Hydrogenophaga* sp. SL48, proteins involved in the biosynthesis of the vancomycin group of antibiotics were present (**Supplementary Tables S13, S14**).

**Figure 1:**
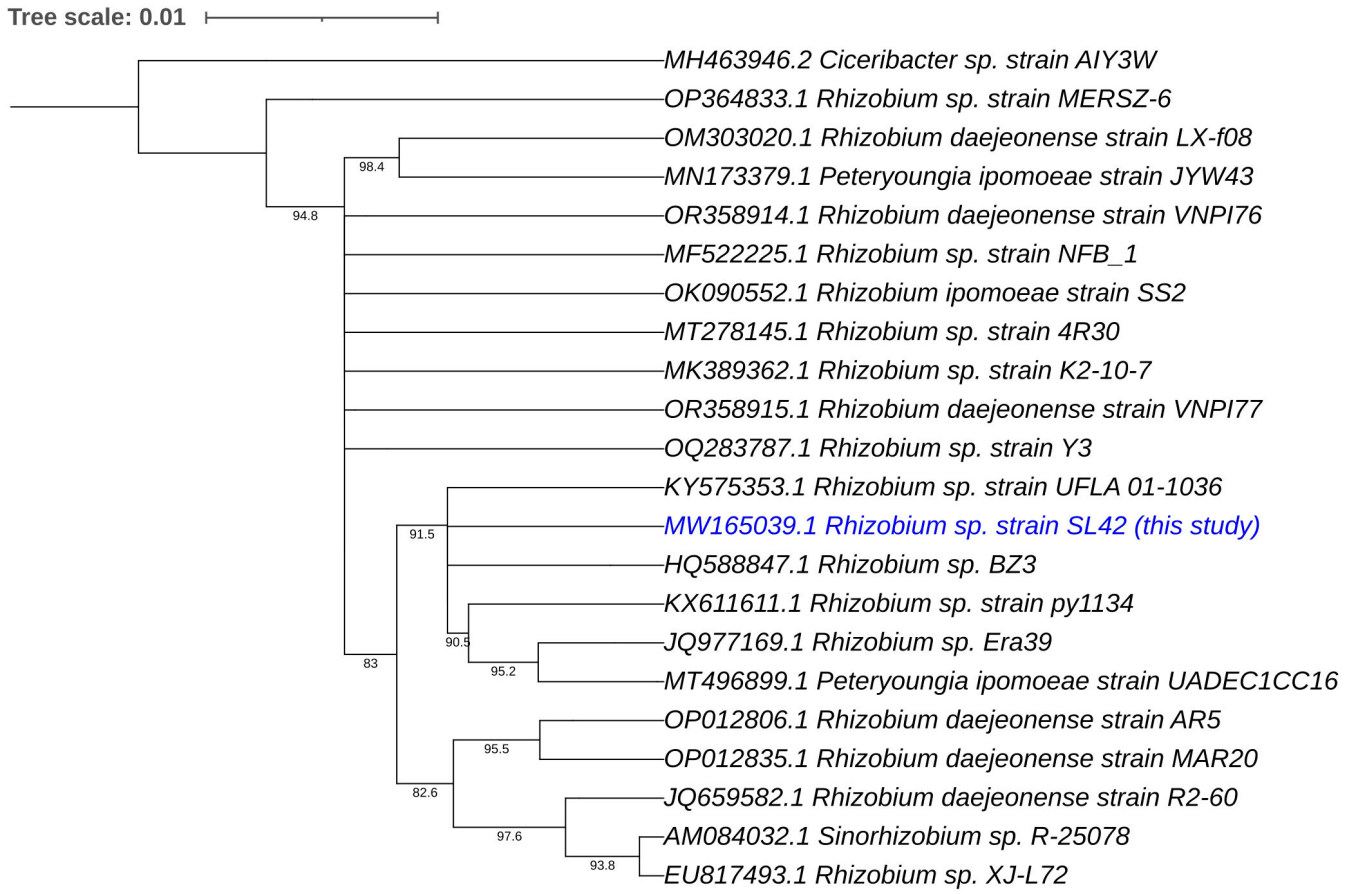
Phylogenetic analysis of *Rhizobium* sp. SL42 (in blue text) based on 16S rRNA gene sequences of related strains. The evolutionary history was inferred using the Maximum Likelihood (ML) method. The bootstrap consensus tree, constructed from 1000 replicate alignments, illustrates the inferred evolutionary history of the taxa analyzed. The percentages displayed next to the branches represent the proportion of these replicate trees in which the associated taxa clustered together, as observed in the bootstrap test. This ML tree was generated employing the Ultrafast Bootstrap analysis and the SH-aLRT branch test. The phylogentic analyses were conducted using IQ-TREE and tree was generated using iTOL.

**Figure 2:**
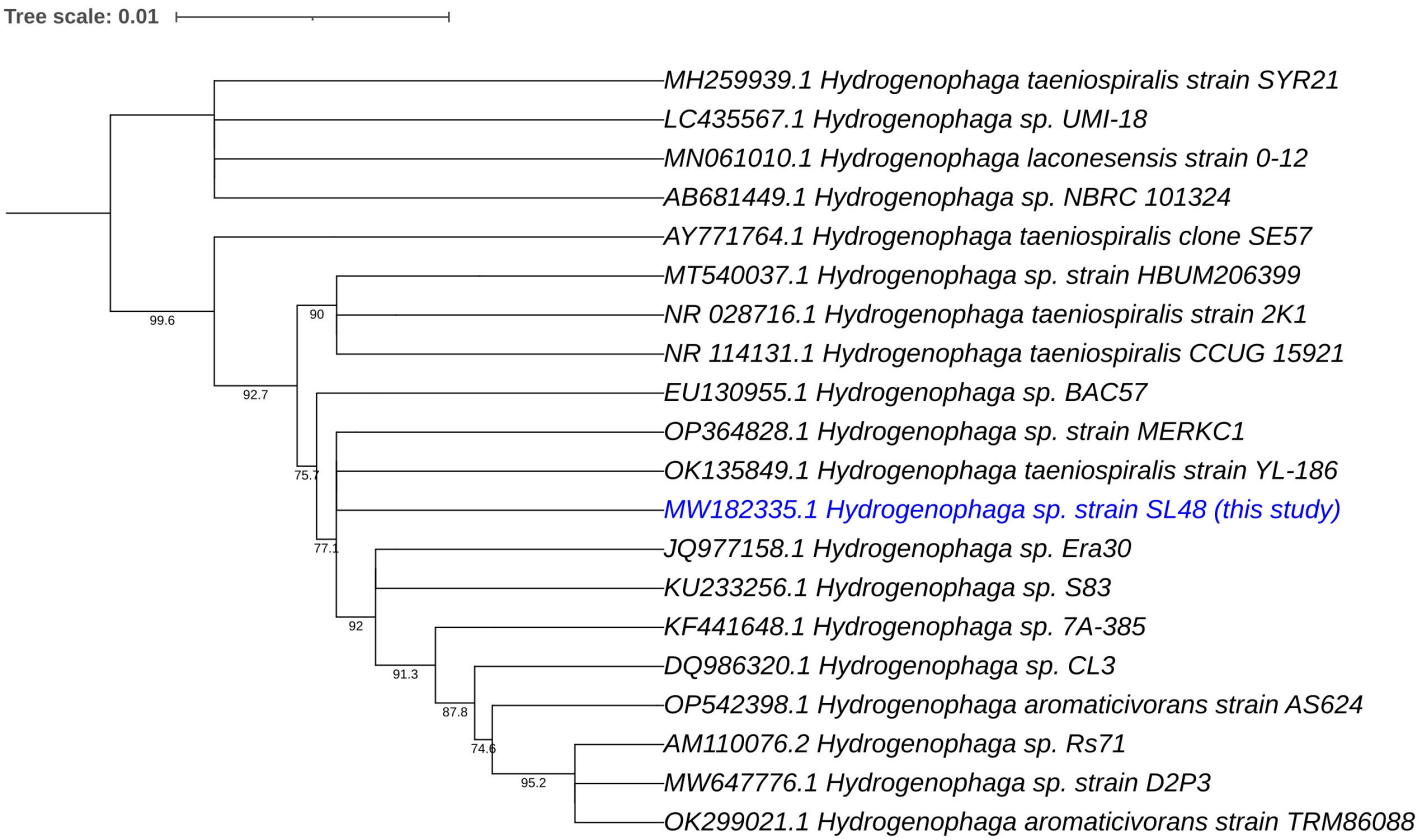
Phylogenetic analysis of *Hydrogenophaga* sp. SL48 (in blue text) based on 16S rRNA gene sequences of related strains. The evolutionary history was inferred using the Maximum Likelihood (ML) method. The bootstrap consensus tree, constructed from 1000 replicate alignments, illustrates the inferred evolutionary history of the taxa analyzed. The percentages displayed next to the branches represent the proportion of these replicate trees in which the associated taxa clustered together, as observed in the bootstrap test. This ML tree was generated employing the Ultrafast Bootstrap analysis and the SH-aLRT branch test. The phylogentic analyses were conducted using IQ-TREE and tree was generated using iTOL.

Phylogenetic analyses of the 16S rRNA gene and house-keeping genes gyrB, recA, and rpoD indicated that *Rhizobium* sp. SL42 and *Hydrogenophaga* sp. SL48 exhibit distinct evolutionary characteristics (**Supplementary Figures S6, S7**). Using 16S rRNA gene sequences of related strains, comprehensive phylogenetic analyses in IQ-TREE enabled a detailed understanding of the evolutionary relationships between them and suggested potential novelty of *Rhizobium* sp. SL42 and *Hydrogenophaga* sp. SL48 within their respective genera, as indicated by distinct branching patterns (**Figures 1**, **2**).

**Figure 3:**
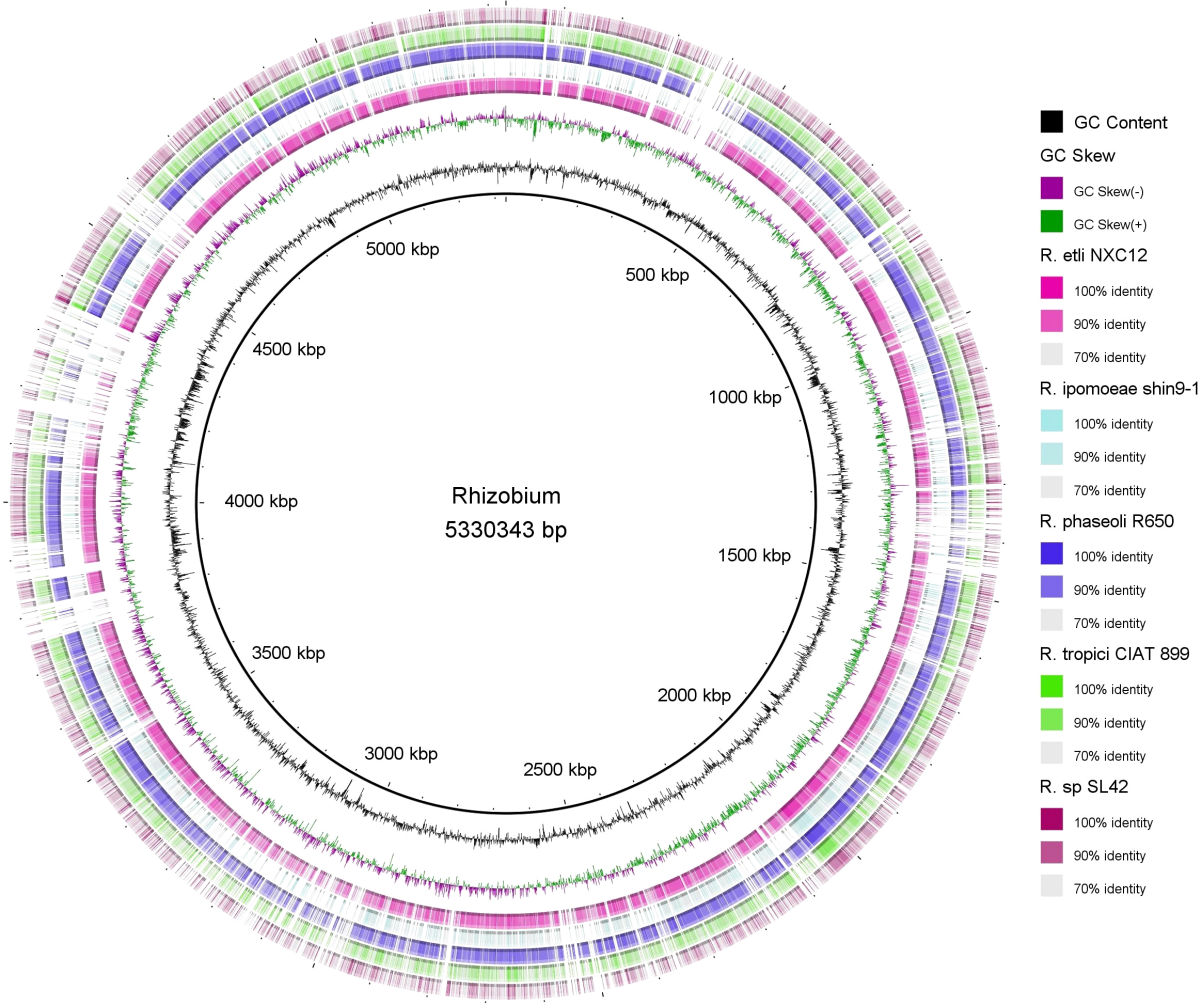
Map of the circular genome of *Rhizobium* sp. SL42, created with CGview, which accentuates the strain’s unique regions and open reading frames (ORFs), and includes BRIG visualizations for comparative genomics. This map facilitates a comparison with other strains within the same genera, highlighting the unique genetic elements that are specific to *Rhizobium* sp. SL42.

**Figure 4:**
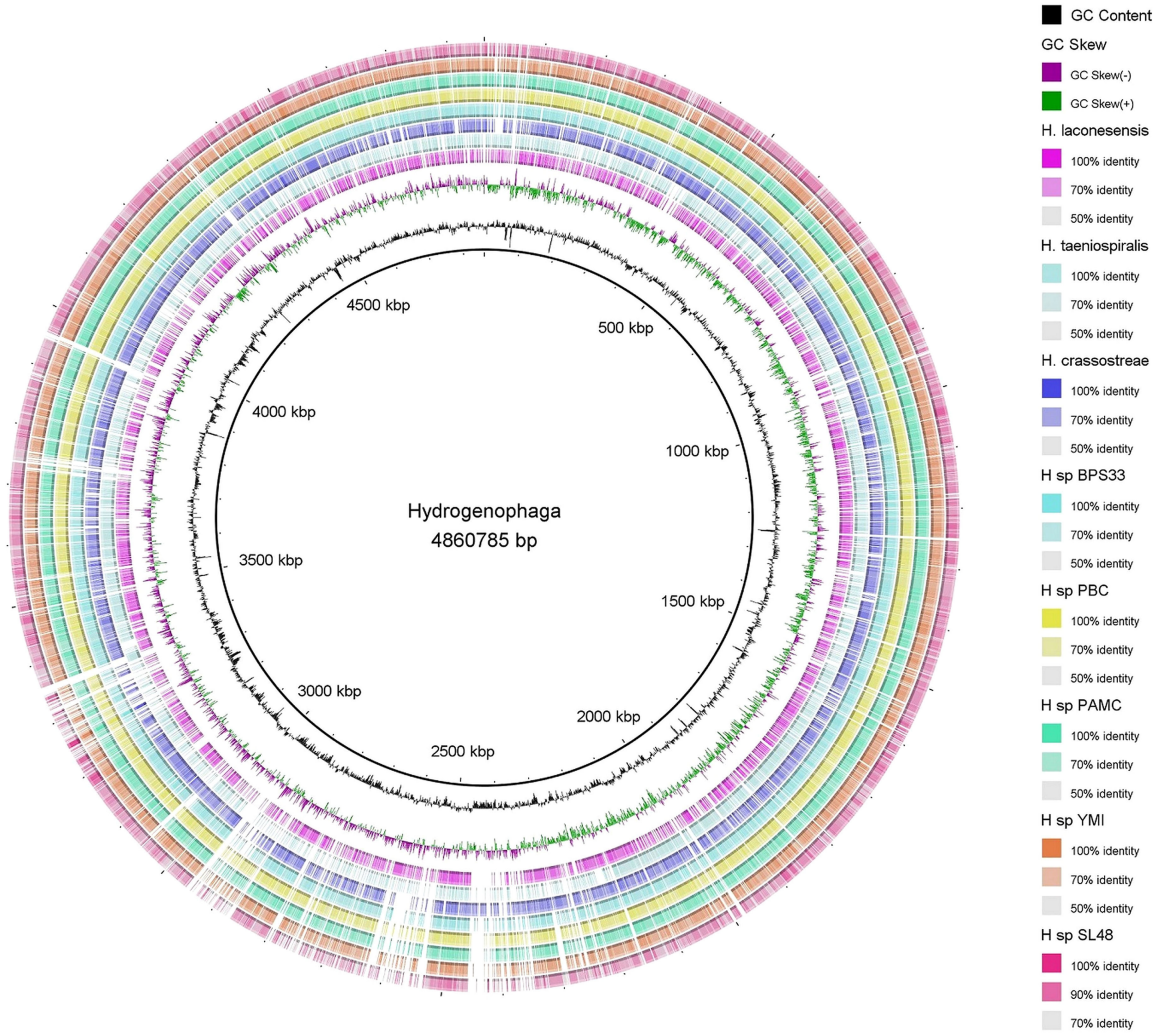
Map of the circular genome of *Hydrogenophaga* sp. SL48, created with CGview, which accentuates the strain’s unique regions and open reading frames (ORFs), and includes BRIG visualizations for comparative genomics. This map facilitates a comparison with other strains within the same genera, highlighting the unique genetic elements that are specific to *Hydrogenophaga* sp. SL48.

Circular genome mapping of *Rhizobium* sp. SL42 and *Hydrogenophaga* sp. SL48 identified unique regions, ORFs, and specific genetic elements exclusive to these strains. These genomic comparisons with other strains within their respective genera highlighted their genetic distinctiveness (**Figures 3**, **4**). M1CR0B1AL1Z3R web server was utilized to analyze genomic features and identify orthologous genes in *Rhizobium* sp. SL42 and *Hydrogenophaga* sp. SL48, comparing them with complete genomes from the NCBI database. The analysis a comprehensive pan-genome analysis covering GC content, ORF distribution, and orthologous group sizes. We focused exclusively on complete and fully assembled genomes to ensure accurate ortholog identification, crucial for studying horizontal gene transfer and bacterial evolution (**Supplementary Data Sheet 1, 2**).

### 3.3 Finding secondary metabolites using Anti-SMASH

The AntiSMASH analysis revealed the presence of biosynthetic gene clusters encoding secondary metabolites in both *Rhizobium* sp. SL42 and *Hydrogenophaga* sp. SL48 strains (**Table 5**). Some of these clusters were unique to each strain. In *Rhizobium* sp. SL42, several regions were identified, including TfuA-related, Terpene, Hserlactone, and Bacteriocin. *Hydrogenophaga* sp. SL48 displayed diverse secondary metabolite coding regions, including Arylpolyene, Terpene, T1PKS, NRPS-like, NRPS, Bacteriocin, Siderophore, and Betalactone. Some of these regions exhibited similarities to known clusters, such as Xanthomonadin I, Desferrioxamine E, and Mycosubtilin (**Supplementary Figures S8, S9**).

**Table 5:** AntiSMASH results of secondary metabolite coding regions of *Rhizobium* sp. SL42 and *Hydrogenophaga* sp. SL48.

*Rhizobium* sp. SL42		*Hydrogenophaga* sp. SL48	
Region	Type	Region	Type
Region 1.1	TfuA-related	Region 1	Arylpolyene
Region 1.2	Terpene	Region 2	Terpene
Region 1.3	Hserlactone	Region 3	T1PKS, NRPS-like, NRPS
Region 1.4	Bacteriocin	Region 4	Bacteriocin
Region 2.1	NRPS, T1PKS	Region 5	Siderophore
Region 2.2	TfuA-related	Region 6	Betalactone
Region 3.1	Hserlactone		

## 4 Discussion

### 4.1 Phylogenetic analysis and genome comparisons

The phylogenetic analyses based on the 16S rRNA gene sequences of related strains facilitated a deeper understanding of the evolutionary dynamics of these bacterial strains and strongly suggested that *Rhizobium* sp. SL42 and *Hydrogenophaga* sp. SL48 might represent novel species within their respective genera. The distinct branching patterns and genetic divergence from known species in their taxonomic groups provide compelling evidence of their uniqueness. The circular genome mapping of these strains revealed unique genomic regions, open reading frames (ORFs), and distinctive elements exclusive to *Rhizobium* sp. SL42 and *Hydrogenophaga* sp. SL48. This demonstrates their genetic distinctiveness and supports the hypothesis that they may represent novel species. The genomic comparisons with other strains within their respective genera highlight these differences.

The use of the M1CR0B1AL1Z3R web server for the computation of key genomic features and identification of orthologous genes is valuable for understanding the genetic relationships of these strains. The identification of orthologous genes can provide insights into the evolutionary history and relatedness of these strains to other known species. The pan genome analysis, which includes aspects such as GC content, ORF distribution, and orthologous group sizes, is essential for gaining a comprehensive understanding of the genetic diversity within the studied bacterial genera. It can help uncover the potential influence of horizontal gene transfer (HGT) events on bacterial evolution, shedding light on how genetic diversity is shaped in these organisms.

### 4.2 Biological significance of genomic features

The genomic analysis has revealed a diverse array of genes associated with various biological functions in both *Rhizobium* sp. SL42 and *Hydrogenophaga* sp. SL48. These features play a crucial role in plant-microbe interactions and plant growth enhancement. The presence of Type I and Type IV secretion systems in *Rhizobium* sp. SL42 is significant because these systems are known to be involved in the communication between rhizobia and leguminous plants (Nelson and Sadowsky, 2015). They play a crucial role in the establishment of symbiotic relationships, leading to effective nodulation and nitrogen fixation. The Type IV secretion system, in particular, is implicated in the transfer of symbiotic signals and genetic material between the bacterium and the plant (Black et al., 2012). This system is essential for the successful formation of nodules on plant roots and the subsequent fixation of atmospheric nitrogen, making it an integral part of the nitrogen fixation process.

Similarly, *Hydrogenophaga* sp. SL48 carries genes for Type II and Type IV secretion systems, which may contribute to interactions with plant roots and environmental adaptation. The presence of hydrogenase enzymes in *Hydrogenophaga* sp. SL48 is also noteworthy. Hydrogenase enzymes are involved in the metabolism of hydrogen gas, which can be a source of electrons for nitrogenase enzymes responsible for nitrogen fixation (Mishra et al., 2019). In nitrogen-fixing bacteria, hydrogenase enzymes play a role in maintaining the redox balance necessary for efficient nitrogen fixation. Therefore, the presence of hydrogenase enzymes in *Hydrogenophaga* sp. SL48 suggests its potential involvement in nitrogen fixation processes.

Additionally, the presence of genes related to photosynthesis, carbon fixation, nitrogen fixation, and carotenoid biosynthesis in *Rhizobium* sp. SL42, and the genes for the biosynthesis of antibiotics in *Hydrogenophaga* sp. SL48, highlight their potential to produce growth-promoting compounds. These genomic features are of significant biological importance as they can contribute to the production of compounds that stimulate plant growth and protect plants from pathogens (Levy et al., 2018).

### 4.3 Ecological and agricultural applications

Plant growth promoting rhizobacteria (PGPR) produce bioactive substances that improve plant growth and alleviate stress. Understanding the behavior of PGPR when inoculated onto plants is important for their application in agriculture. Some of these compounds are also essential for plant root colonization (Bloemberg and Lugtenberg, 2001). The whole genome sequencing analysis revealed the genes harbored in the genomes of *Rhizobium* sp. SL42 and *Hydrogenophaga* sp. SL48 that might play key roles in plant-microbe interactions. PGPR are known to produce auxins, gibberellins, cytokinins and ethylene and manipulate phytohormone balance in plants. PGPR stimulate root proliferation by excretion of indole-3-acetic acid (IAA) into the rhizosphere, thus enhancing uptake of water and nutrients (Sukumar et al., 2013). Several PGPR also secrete cytokinins that have been detected in cell free medium (Garcia de Salamone et al., 2001). Genes encoding IAA and cytokinin biosynthesis (*iaaM* and *fas6*) were present in *Hydrogenophaga* sp. SL48. Volatile organic compounds (VOCs) produced by bacteria help in plant development and stress responses (Bailly and Weisskopf, 2012). Polyamines play important physiological and protective roles in plants. *Bacillus megaterium* BOFC15 secretes spermidine, a polyamine leading to enhanced cellular polyamine levels in Arabidopsis. Inoculation with the bacterium resulted in an increase in biomass, changed root architecture and elevated photosynthetic capacity. The plants also exhibited higher drought tolerance and abscisic acid content under water deficit (osmotic stress) (Zhou et al., 2016a). Both strains possess multiples genes that encode for spermidine/putrescine compounds (potA, osmY, envZ). These genomic findings have implications for potential ecological and agricultural applications. Understanding the genomic features of these strains can lead to the development of more effective PGPR inoculants for agricultural use.

Genes encoding the production of secondary metabolites found using Anti-SMASH showed that the PGPR produces antibiotics such as thiopeptides, polyketides and bacteriocins that suppress pathogens. These findings underscore the potential of these strains to produce bioactive compounds, further contributing to their functional properties in plant growth stimulation and ecosystem interactions reported in our previous studies (Ilangumaran et al., 2022). Bacterial surface factors like flagellins and o-antigen of lipopolysaccharides induce systemic resistance (ISR) whereas, analogs of salicylic acid, jasmonic acid and ethylene elicit systemic acquired resistance (SAR) in plants (Ping and Boland, 2004; Lugtenberg and Kamilova, 2009; Pieterse et al., 2014). A bacteriocin, thuricin 17, isolated from the soybean endosymbiont *Bacillus thuriengenesis* NEB 17, when applied as foliar spray or root drench stimulated the growth of soybean and corn (Subramanian et al., 2016). Siderophores are iron chelators produced by some microorganisms and enhance plant growth under iron-depleted conditions where they are used as the method for accessing scarce iron and also act as biocontrol agents by reducing the availability of iron for pathogens (Saha et al., 2016). The identified biosynthetic gene clusters for antibiotics, polyketides, and bacteriocins might suppress plant pathogens, thus contributing to plant health and growth.

### 4.4 Rhizosphere colonization

Genes involved in the pathways of cell motility, chemotaxis, lipopolysaccharide synthesis and biofilm formation suggested that they might play important roles in rhizosphere colonization of *Rhizobium* sp. SL42 and *Hydrogenophaga* sp. SL48. Plant beneficial bacteria present in the rhizosphere are in proximity to roots and many are known to form biofilms, which aid in the successful colonization of root surfaces and adjacent soil particles and thwart pathogenic bacteria. Biofilms are structured communities of bacterial cells living adherent to a surface embedded in an extracellular polysaccharide matrix. Biofilms of beneficial bacteria play a crucial role in plant growth promoting effects, as they aid in the colonization of root surfaces and the inhibition of pathogenic bacteria (Ramey et al., 2004). Plant roots exude signal compounds that regulate plant-bacteria interactions and trigger chemotaxis in bacteria, towards the rhizosphere (Fan et al., 2012). For example, flavonoids secreted by roots determine the legume-rhizobia symbiotic associations while malate and citrate are found to interact with *Bacillus* and *Pseudomonas* strains (Badri and Vivanco, 2009). There are genes related to the metabolism of these compounds in *Rhizobium* sp. SL42 and *Hydrogenophaga* sp. SL48, suggesting that they possibly take part in plant-microbe interactions.

During colonization, PGPR assimilate substances released by the roots and in turn, produce bioactive compounds that promote plant growth or ameliorate stress (Xie et al., 2014). Recent advances in high-throughput strategies have led to detailed investigations of plant-microbe interactions and the differential effects of root exudates on mechanisms of rhizobacteria that are crucial to the beneficial effects observed. The differentially expressed genes or proteins were mainly those involved in nutrient utilization and transport, chemotaxis, secretion, quorum sensing, extracellular matrix, synthesis of volatile compounds, and antibiotic production (Fan et al., 2012; Beauregard et al., 2013; Kierul et al., 2015; Mwita et al., 2016; Zhou et al., 2016b). The presence of genes related to the metabolism of root-exuded compounds, such as flavonoids and organic acids, in these strains indicates their ability to respond to plant signals and interact with the rhizosphere. This is essential for establishing beneficial relationships with plants and enhancing nutrient uptake.

Understanding the dynamic function of bacterial cells and regulatory networks related to enzyme metabolism, transport and utilization of nutrients, signal transduction proteins and root colonization pattern is important in determining their potential applications in agriculture (Kierul et al., 2015; Zhang et al., 2015; Mwita et al., 2016; Zhou et al., 2016b). The genomic features in *Rhizobium* sp. SL42 and *Hydrogenophaga* sp. SL48 hold great potential for enhancing their ability to establish beneficial relationships with soybeans. These features, including nutrient acquisition, hormone production, and bioactive compound production, are of clear biological significance in the context of plant-microbe interactions and growth enhancement. This suggests promising applications for these strains in agriculture to improve crop productivity and promote sustainable farming practices.

## 5 Summary

Genomic analysis of *Rhizobium* sp. SL42 and *Hydrogenophaga* sp. SL48 revealed the presence of genes encoding various metabolic functions and compound synthesis. These strains were selected for sequencing as part of the isolation and characterization of beneficial rhizobacteria from native relatives of cultivated plants, chosen based on their ecological and agricultural importance in plant-microbe interactions, stress tolerance, and growth promotion. The sequencing aimed to determine their plant growth promoting and genetic characteristics. The findings highlight the functional diversity of these bacterial strains and suggest their potential roles in ecological interactions and adaptation strategies. The presence of essential genes associated with plant-microbe interactions, along with unique proteins, presents promising avenues for exploring their contributions to plant growth stimulation and antibiotic production. The study enriches our understanding of microbial diversity and potential biotechnological applications.

The combined results of biosynthetic gene cluster analyses, phylogenetic assessments, and genomic mapping provide compelling evidence for the novelty of *Rhizobium* sp. SL42 and *Hydrogenophaga* sp. SL48 within their respective genera. Unique biosynthetic gene clusters, distinct phylogenetic positions, and exclusive genomic regions further support their potential classification as entirely new species. These findings underscore the significance of these bacterial strains in terms of microbial diversity and their potential contributions to plant growth stimulation and ecological interactions. The discovery of novel bacterial species enhances our understanding of microbial ecology and opens promising avenues for utilizing the beneficial properties of these bacteria in agriculture and the environment.

## Data availability statement

The datasets presented in this study can be found in online repositories. The names of the repository/repositories and accession number(s) can be found in the article/**Supplementary Material**.

## Author contributions

GI: Conceptualization, Data curation, Formal analysis, Investigation, Methodology, Project administration, Resources, Software, Validation, Visualization, Writing – original draft. SS: Conceptualization, Data curation, Formal analysis, Funding acquisition, Investigation, Methodology, Project administration, Resources, Software, Supervision, Validation, Visualization, Writing – original draft, Writing – review & editing. DS: Formal Analysis, Funding acquisition, Investigation, Project administration, Resources, Supervision, Writing – review & editing.
