# Supplementary material for: Complete genome sequences of Rhizobium sp. strain SL42 and Hydrogenophaga sp. strain SL48, microsymbionts of Amphicarpaea bracteata
Source: Front Microbiomes. 2024 Feb 13;3:1309947. doi: 10.3389/frmbi.2024.1309947 (PMC12993520; doi:10.3389/frmbi.2024.1309947)
Supplement: Supplementary file 3 [file Presentation_1.pdf]

Supplementary Material

1 Supplementary Figures

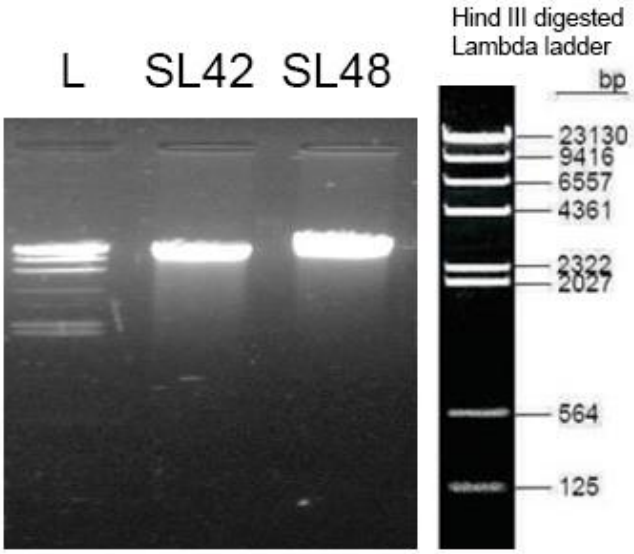

Supplementary Figure S1. Agarose gel electrophoresis of DNA samples.

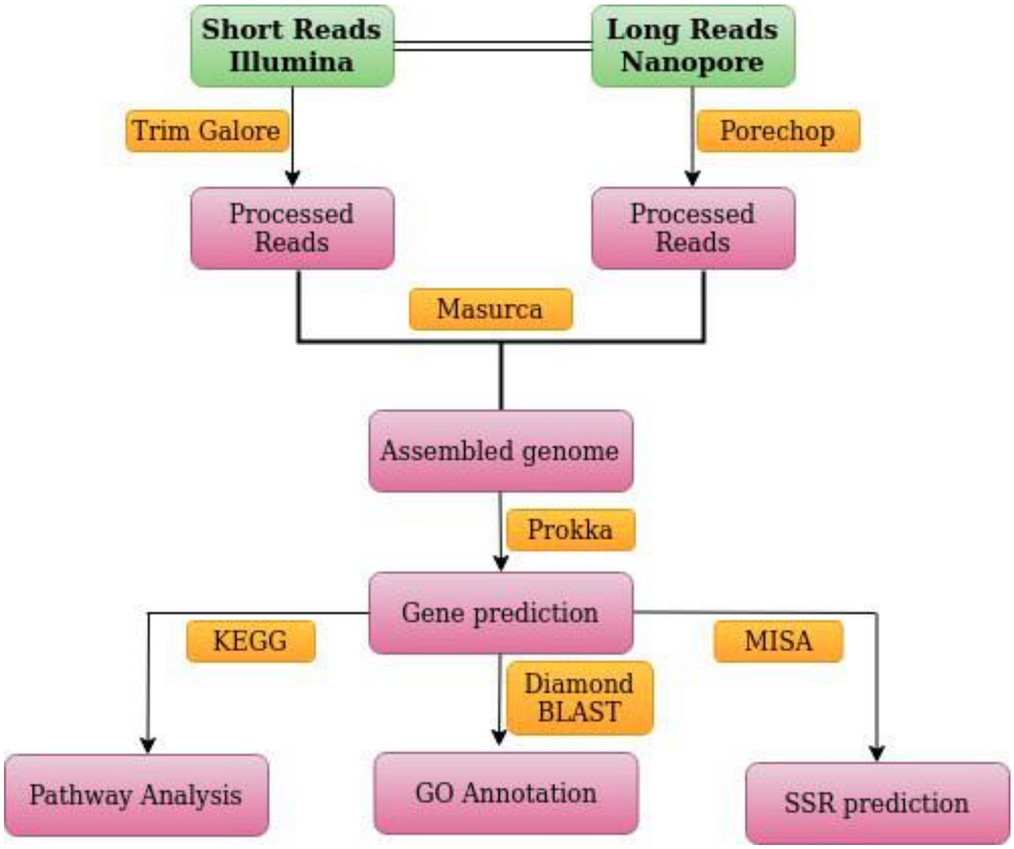

Supplementary Figure S2. Methodology for *de novo* genome sequence analysis

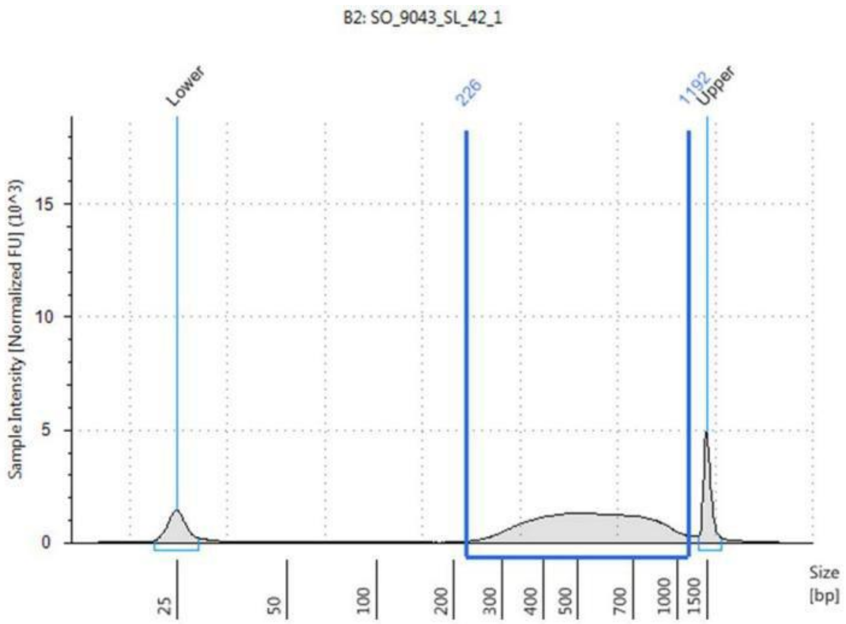

From [bp]	To [bp]	Average Size [bp]	Conc. [ng/µl]	Region Molarity [nmol/l]	% of Total
226	1192	574	28.9	86.7	95.27

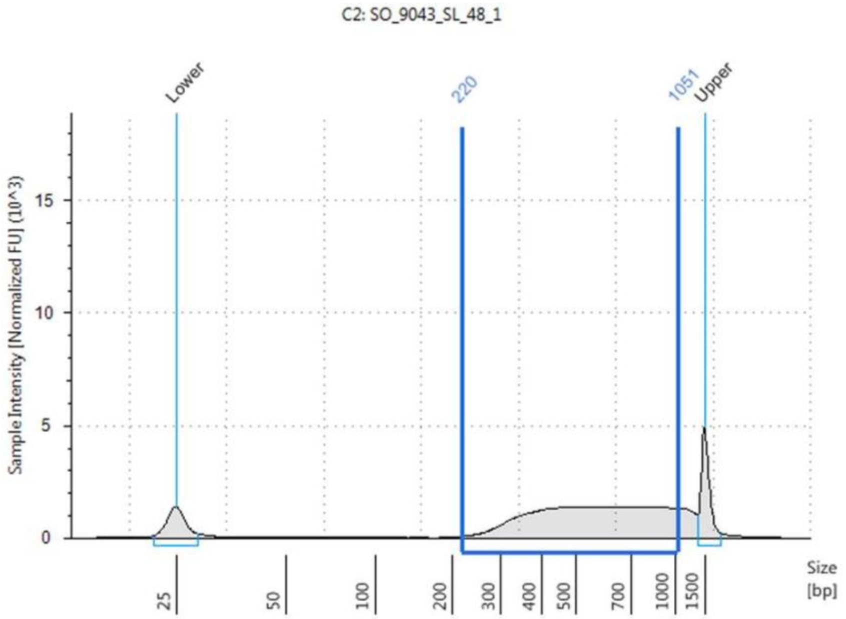

From [bp]	To [bp]	Average Size [bp]	Conc. [ng/µl]	Region Molarity [nmol/l]	% of Total
220	1051	591	31.1	91.9	88.33

Supplementary Figure S3. TapeStation Profiles of SL42 and SL48 DNA libraries

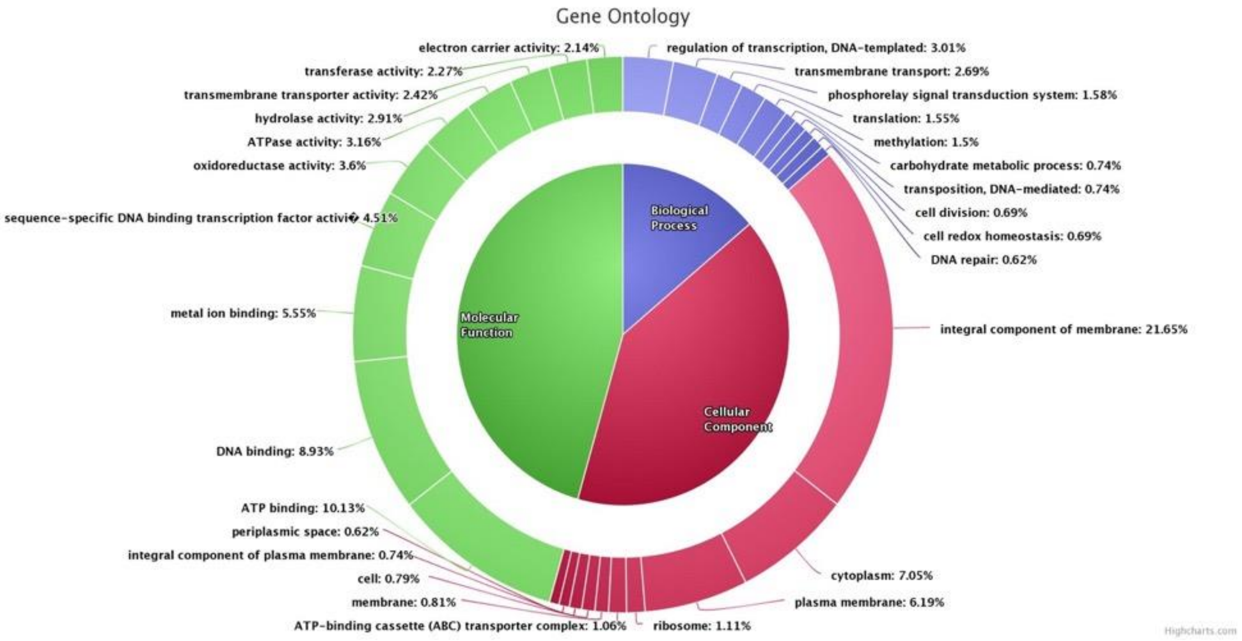

Supplementary Figure S4. Gene Ontology distribution of annotated proteins in *Rhizobium* sp. SL42 genome

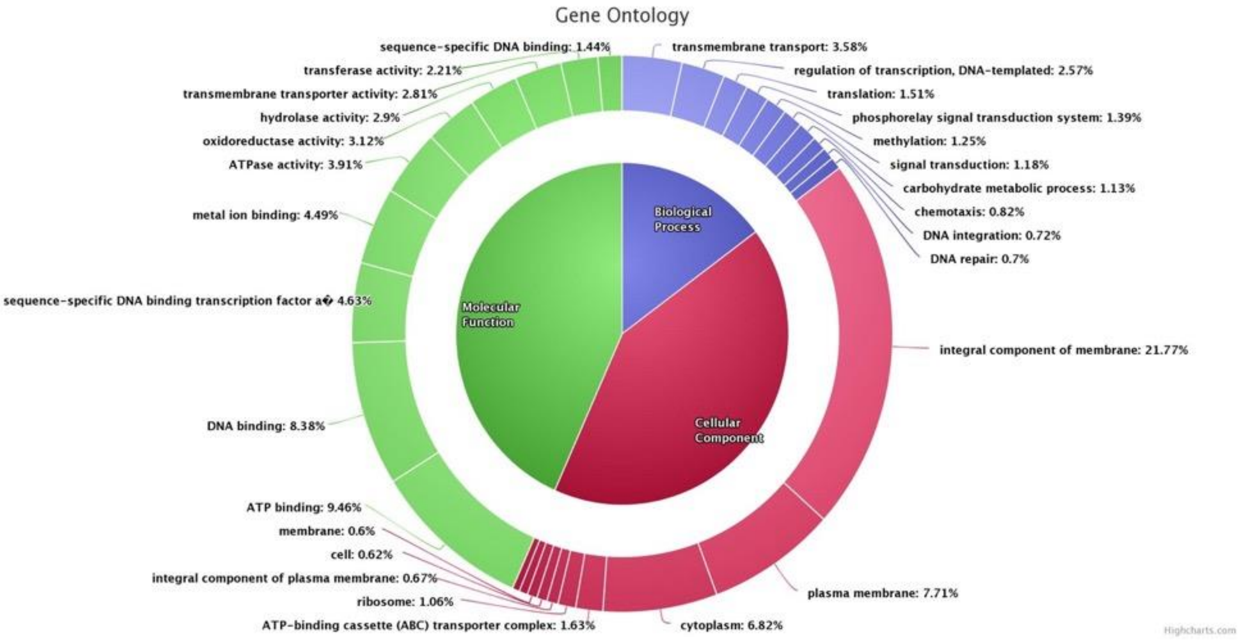

Supplementary Figure S5. Gene Ontology distribution of annotated proteins in *Rhizobium* sp. SL42 genome

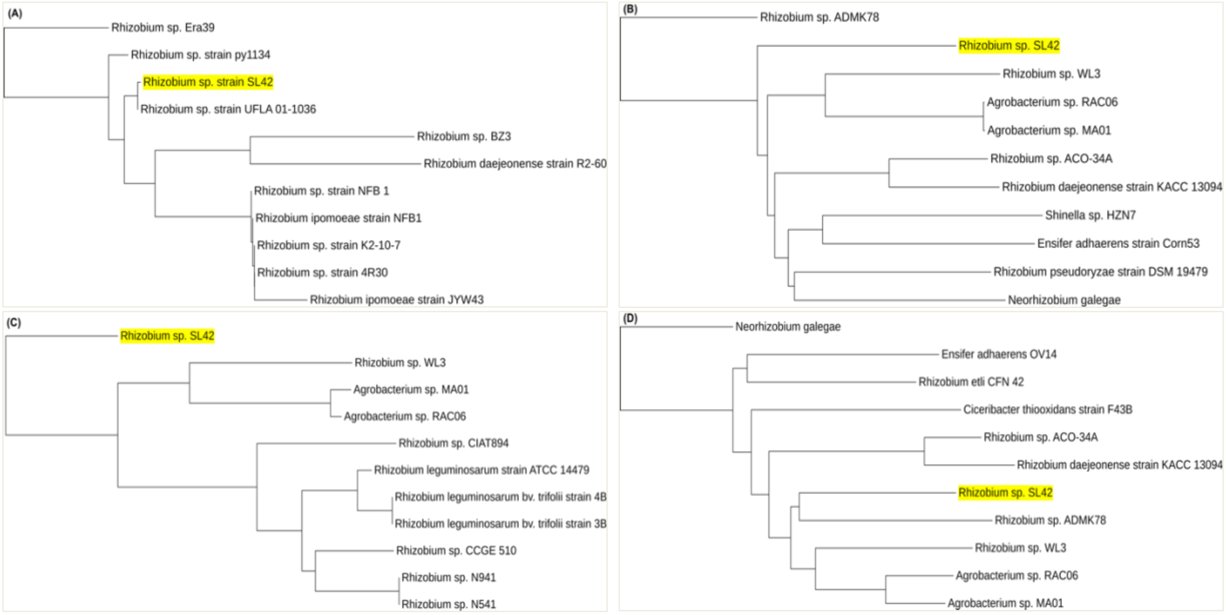

Supplementary Figure S6. Phylogenetic trees of *Rhizobium sp.* SL42 using BLAST pairwise alignment. Query gene sequences (A) *16S* rRNA (B) *gyrB* (C) *recA* and (D) *rpoD*.

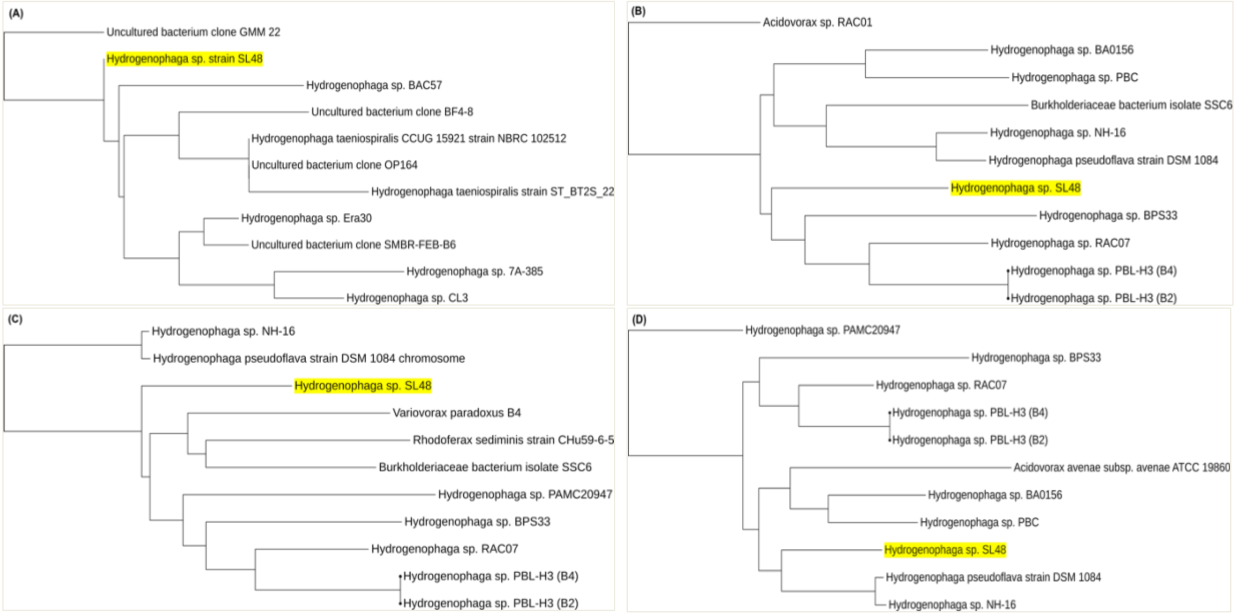

Supplementary Figure S7. Phylogenetic trees of *Hydrogenophaga sp.* SL48 using BLAST pairwise alignment. Query gene sequences (A) *16S* rRNA (B) *gyrB* (C) *recA* and (D) *rpoD*.

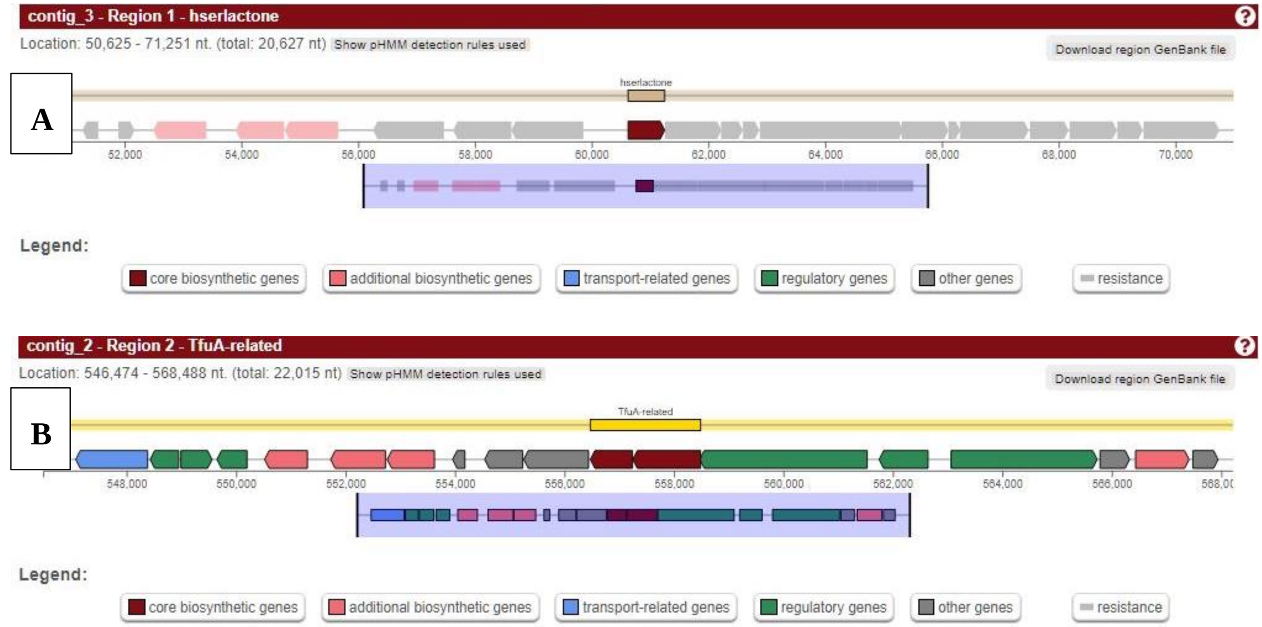

Supplementary Figure S8. Coding regions of (A) homoserine lactone and (B) TfuA-related in SL42 genome

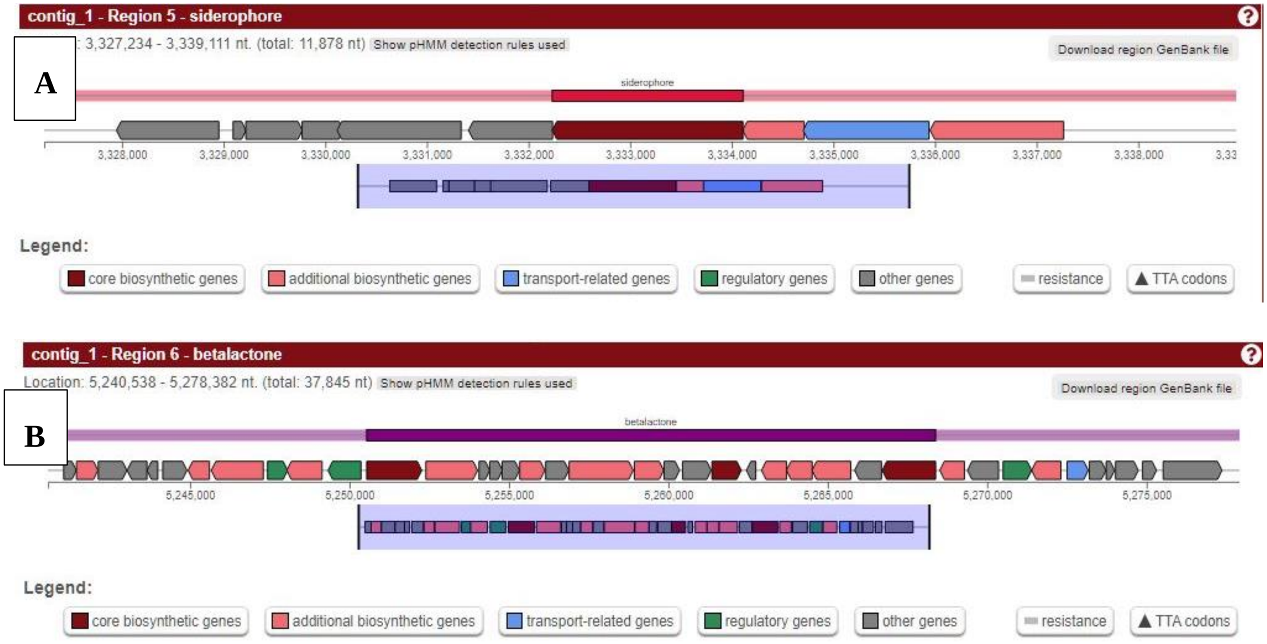

Supplementary Figure S9. Coding regions of (A) siderophore and (B) betalactone in SL48 genome

2 Supplementary Tables

Supplementary Table S1. DNA Concentration and Purity of samples estimated using Nanodrop Spectrophotometer and Qubit Flurometer

	Sample	SL42_1	SL48_1
Nanodrop	ng/µL	456.4	910.7
	260/280	1.89	1.93
	260/230	1.67	1.97
	Volume (µL)	25	25
	Yield (ng)	11410	22767.5
	Volume loaded on gel (ng/µL)	2 (1:4)	2 (1:7)
Qubit QC	Qubit conc. (ng/µL)	391.2	936.6
	Volume (µL)	25	25
	Yield (ng)	9780	23415
	QC purity	Optimal	Optimal
	QC Integrity	Intact	Intact

Supplementary Table S2. Primer Sequences

16S rRNA primers		
27F’	AGAGTTTGATCCTGGCTCAG	
1492R’	TACGGCTACCTTGTTACGACTT	
**Illumina Adapter details**		
Universal Adapter	5’AATGATACGGCGACCACCGAGATCTACACTCTTTCCCTACAC GACGCTCTTCCGATCT	
Adapter, Index	5’GATCGGAAGAGCACACGTCTGAACTCCAGTCAC [INDEX] ATCTCGTATGCCGTCTTCTGCTTG	
**Barcodes used for Nanopore sequencing**		
Sample ID	Barcode name	Sequences
SL42_1	NB05	AAGGTTACACAAACCCTGGACAAG
SL48_1	NB06	GACTACTTTCTGCCTTTGCGAGAA

Supplementary Table S3. Description of libraries

Sample ID	SL42_1	SL48_1
Qubit Conc. (ng/µl))	25.2	37
Volume (µl)	10	10
Yield (ng)	252	370
Index 1	N704	N705
Index 1 Sequence	TCCTGAGC	GGACTCCT
Index 2	S502	S502
Index 2 Sequence	CTCTCTAT	CTCTCTAT

Supplementary Table S4. Tapestation Profile of the libraries

	SL42	SL48
From (bp)	226	220
To (bp)	1192	1051
Average (bp)	574	591
Conc. ng/ml	28.9	31.1
Region Molarity nmol/l	86.7	91.9

Supplementary Table S5. FastQC output on raw sequence data

Measure	SL42	SL48
File type	Conventional base calls	Conventional base calls
Encoding	Sanger/Illumina 1.9	Sanger/Illumina 1.9
Total sequences	2782606	3022451
Sequences flagged as poor quality	0	0
Sequence length (bp)	150	150
% GC	60	65

Supplementary Table S6. Illumina read statistics

**Sample**	**Raw Read**	**Processed Reads**	**% Reads Retained**
SL42	2782606	2533621	91.05
SL48	3022451	2780700	92.00

Supplementary Table S7. Nanopore read statistics

**Statistics**	**SL42_1**	**SL48_1**
Reads Generated	60262	44739
Maximum Read Length	71443	91232
Minimum Read Length	19	38
Average Read Length	6311.1	7323.4
Median Read Length	271.5	3762
Total Reads Length	380317539	327639793
Reads >= 100 bp	60213	44712
Reads >= 200 bp	58663	43957
Reads >= 500 bp	49279	40114
Reads >= 1 Kbp	44337	36607
Reads >= 10 Kbp	13605	11252
N50 value	11838	13578

Supplementary Table S8. Read statistics combined

	SL42_1_barcode05		SL48_1_barcode06	
**Nanopore combined**	**Raw**	**Processed**	**Raw**	**Processed**
Contigs Generated	110664	60262	82669	44739
Maximum Contig Length	71508	71443	91305	91232
Minimum Contig Length	117	19	121	38
Average Contig Length	5771.1	6311.1	6966.1	7323.4
Median Contig Length	672.5	271.5	3972	3762
Total Contigs Length	638654847	380317539	575884066	327639793
Total Number of Non-ATGC	0	0	0	0
% of Non-ATGC Characters	0	0	0	0
Contigs >= 100 bp	110664	60213	82669	44712
Contigs >= 200 bp	110218	58663	82354	43957
Contigs >= 500 bp	88956	49279	74407	40114
Contigs >= 1 Kbp	75713	44337	66310	36607
Contigs >= 10 Kbp	22414	13605	19423	11252
N50 value	11575	11838	13208	13578

Supplementary Table S9. Sequencing coverage

Sample	SL42	SL48
Illumina	166.96	181.35
Nanopore	127.73	115.18

Supplementary Table S10. Assembly statistics

**Assembly statistics**	**SL42**	**SL48**
Contigs Generated	3	1
Maximum Contig Length	4063937	5433040
Minimum Contig Length	351829	5433040
Average Contig Length	1722001	5433040
Median Contig Length	750237.0	5433040.0
Total Contigs Length	5166003	5433040
Contigs >= 10 Kbp	3	1
Contigs >= 1 Mbp	1	1
N50 value	4063937	5433040

Supplementary Table S11. Cut-offs and range of SSR in the genome assembly

Statistics	SL42	SL48
Total number of sequences examined	3	1
Total size of examined sequences (bp)	5166003	5433040
Total number of identified SSRs	116	153
Number of SSR containing sequences	3	1
Number of compound SSRs	1	1
Mono nucleotide repeats p1 >= 10 bases	1	8
Di nucleotide repeats p2 >= 6 Pairs	4	26
Tri nucleotide repeats p3 >= 5 Sets	10	17
Tetra nucleotide repeats p4 >= 3 Sets	100	99
Penta nucleotide repeats p5 >= 5 Sets	1	1
Hexa nucleotide repeats p6 >= 5 Sets	3	2

Supplementary Table S12. Annotation summary of predicted proteins

Sample	Total proteins	Annotated proteins
SL42	4727	4642
SL48	5077	4937

Supplementary Table S13. KEGG pathway analysis of predicted proteins in SL42 genome

Pathway function	Pathway
BRITE hierarchy	
Genetic information processing	Transcription factors, Translation factors, Transcription machinery, tRNA biogenesis, mRNA biogenesis<br>Mitochondrial biogenesis, Ribosome biogenesis, Ribosome proteins<br>DNA replication proteins, DNA repair and recombination proteins<br>Chromosome and associated proteins, Chaperones and folding catalysts, Membrane trafficking
Metabolism	Photosynthesis proteins<br>Amino acid related enzymes, Protein kinases, Protein phosphatases and associated proteins, Peptidases and inhibitors<br>Peptidoglycan biosynthesis and degradation proteins, Lipopolysaccharide biosynthesis, Lipid biosynthesis proteins<br>Glycosyltransferases, Prenyltransferases
Signaling and cellular processes	Antimicrobial resistance genes, Prokaryotic defense system, Exosome<br>Transporters, Two-component system, Cytoskeleton proteins<br>Bacterial motility proteins, Bacterial toxins, Secretion system
Cellular processes	
Cell motility	Bacterial chemotaxis, Flagellar assembly
Cellular community	Quorum sensing
Environmental information processing	
Membrane transport	ABC transporters<br>Phosphotransferase system (PTS)<br>Bacterial secretion system
Signal transduction	Two-component system
Genetic Information Processing	
Folding, sorting and degradation	RNA degradation<br>Protein export<br>Sulfur relay system
Replication and repair	DNA replication, Homologous recombination<br>Base excision repair, Nucleotide excision repair, Mismatch repair
Transcription	RNA polymerase
Translation	Aminoacyl-tRNA biosynthesis<br>Ribosome proteins
Human diseases	
Anti-microbial drug resistance	Beta-lactam resistance<br>Vancomycin resistance<br>Cationic antimicrobial peptide (CAMP) resistance
Metabolism	
Amino acid	Arginine biosynthesis<br>Alanine, aspartate and glutamate metabolism<br>Glycine, serine and threonine metabolism<br>Cysteine and methionine metabolism<br>Valine, leucine and isoleucine degradation<br>Valine, leucine and isoleucine biosynthesis<br>Lysine biosynthesis, Lysine degradation

	Arginine and proline metabolism<br>Histidine metabolism, Tyrosine metabolism<br>Phenylalanine metabolism, Tyrosine metabolism<br>Tryptophan biosynthesis
Secondary metabolites	Monobactam biosynthesis, Carbapenem biosynthesis<br>Penicillin and cephalosporin biosynthesis, Prodigiosin biosynthesis<br>Novobiocin biosynthesis, Streptomycin biosynthesis<br>Neomycin, kanamycin and gentamicin biosynthesis
Carbohydrate metabolism	Glycolysis / Gluconeogenesis, TCA cycle<br>Pentose phosphate pathway, Pentose and glucuronate interconversions<br>Fructose and mannose metabolism, Galactose metabolism<br>Ascorbate and aldarate metabolism<br>Starch and sucrose metabolism<br>Amino sugar and nucleotide sugar metabolism<br>Inositol phosphate metabolism, Pyruvate metabolism<br>Glyoxylate and dicarboxylate metabolism<br>Propanoate metabolism, Butanoate metabolism, C5-Branched dibasic acid metabolism
Energy metabolism	Oxidative phosphorylation, Methane metabolism<br>Carbon fixation in photosynthetic organisms<br>Nitrogen metabolism, Sulfur metabolism
Glycan biosynthesis and metabolism	Lipopolysaccharide biosynthesis<br>Peptidoglycan biosynthesis, Other glycan degradation
Lipid metabolism	Fatty acid biosynthesis, Fatty acid degradation, Biosynthesis of unsaturated fatty acids Glycerolipid metabolism, Glycerophospholipid metabolism<br>Synthesis and degradation of ketone bodies
Metabolism of cofactors and vitamins	Ubiquinone and other terpenoid-quinone biosynthesis<br>One carbon pool by folate metabolism, Folate biosynthesis<br>Thiamine metabolism, Riboflavin metabolism, Vitamin B6 metabolism<br>Nicotinate and nicotinamide metabolism, Biotin metabolism<br>Pantothenate and CoA biosynthesis, Lipoic acid metabolism<br>Porphyrin and chlorophyll metabolism
Metabolism of other amino acids	beta-Alanine metabolism, D-Alanine metabolism<br>Taurine and hypotaurine metabolism, Phosphonate and phosphinate metabolism<br>Selenocompound metabolism, Cyanoamino acid metabolism<br>D-Glutamine and D-glutamate metabolism, Glutathione metabolism
Metabolism of terpenoids and polyketides	Geraniol degradation, Limonene and pinene degradation<br>Terpenoid backbone biosynthesis<br>Carotenoid biosynthesis<br>Biosynthesis of type II polyketide products
Nucleotide metabolism	Purine metabolism<br>Pyramidine metabolism
Xenobiotics biodegradation and metabolism	Chlorocyclohexane and chlorobenzene degradation<br>Benzoate degradation, Fluorobenzoate degradation, Aminobenzoate degradation, Dioxin degradation, Xylene degradation

	Toluene degradation, Nitrotoluene degradation, Naphthalene degradation, Chloroalkane and chloroalkene degradation<br>Polycyclic aromatic hydrocarbon degradation, Styrene, Atrazine, Caprolactam degradation

Supplementary Table S14. KEGG pathway analysis of predicted proteins in SL48 genome

Pathway function	Pathway
BRITE hierarchy	
Genetic information processing	Transcription factors, Translation factors, Transcription machinery, tRNA biogenesis, mRNA biogenesis<br>Mitochondrial biogenesis, Ribosome biogenesis, Ribosome proteins<br>DNA replication proteins, DNA repair and recombination proteins<br>Chromosome and associated proteins, Chaperones and folding catalysts, Membrane trafficking
Metabolism	Photosynthesis proteins<br>Amino acid related enzymes, Protein kinases, Protein phosphatases and associated proteins, Peptidases and inhibitors<br>Peptidoglycan biosynthesis and degradation proteins, Lipopolysaccharide biosynthesis, Lipid biosynthesis<br>Glycosyltransferases, Prenyltransferases
Signaling and cellular processes	Antimicrobial resistance genes, Prokaryotic defense system, Exosome<br>Transporters, Two-component system, Cytoskeleton proteins<br>Bacterial motility proteins, Bacterial toxins, Secretion system
Cellular processes	
Cell motility	Bacterial chemotaxis, Flagellar assembly
Cellular community	Quorum sensing
Environmental information processing	
Membrane transport	ABC transporters<br>Bacterial secretion system
Signal transduction	Two-component system
Genetic Information Processing	
Folding, sorting and degradation	RNA degradation<br>Protein export<br>Sulfur relay system
Replication and repair	DNA replication, Homologous recombination<br>Base excision repair, Nucleotide excision repair, Mismatch repair
Transcription	RNA polymerase
Translation	Aminoacyl-tRNA biosynthesis<br>Ribosome proteins
Human diseases	
Anti-microbial drug resistance	Beta-lactam resistance<br>Vancomycin<br>Cationic antimicrobial peptide (CAMP) resistance
Metabolism	
Amino acid	Arginine biosynthesis<br>Alanine, aspartate and glutamate metabolism<br>Glycine, serine and threonine metabolism

	Cysteine and methionine metabolism<br>Valine, leucine and isoleucine degradation<br>Valine, leucine and isoleucine biosynthesis<br>Lysine biosynthesis, Lysine degradation<br>Arginine and proline metabolism<br>Histidine metabolism, Tyrosine metabolism<br>Phenylalanine, tyrosine and tryptophan biosynthesis
Secondary metabolites	Monobactam biosynthesis, Carbapenem biosynthesis<br>Novobiocin biosynthesis, Phenazine biosynthesis<br>Streptomycin biosynthesis<br>Neomycin, kanamycin and gentamicin biosynthesis<br>Acarbose and validamycin biosynthesis
Carbohydrate metabolism	Glycolysis / Gluconeogenesis, TCA cycle<br>Pentose phosphate pathway, Pentose and glucuronate interconversions<br>Fructose and mannose metabolism, Galactose metabolism<br>Ascorbate and aldarate metabolism<br>Starch and sucrose metabolism<br>Amino sugar and nucleotide sugar metabolism<br>Inositol phosphate metabolism, Pyruvate metabolism<br>Glyoxylate and dicarboxylate metabolism<br>Propanoate metabolism, Butanoate metabolism, C5-Branched dibasic acid metabolism
Energy metabolism	Oxidative phosphorylation, Methane metabolism, Nitrogen metabolism, Sulfur metabolism
Glycan biosynthesis and metabolism	Lipopolysaccharide biosynthesis<br>Peptidoglycan biosynthesis
Lipid metabolism	Fatty acid biosynthesis, Glycerolipid metabolism
Metabolism of cofactors and vitamins	Ubiquinone and other terpenoid-quinone biosynthesis<br>One carbon pool by folate metabolism, Folate biosynthesis<br>Thiamine metabolism, Riboflavin metabolism, Vitamin B6 metabolism<br>Nicotinate and nicotinamide metabolism, Biotin metabolism<br>Pantothenate and CoA biosynthesis, Lipoic acid metabolism<br>Porphyrin and chlorophyll metabolism
Metabolism of other amino acids	beta-Alanine metabolism, D-Alanine metabolism<br>Selenocompound metabolism, Cyanoamino acid metabolism<br>D-Glutamine and D-glutamate metabolism, Glutathione metabolism
Metabolism of terpenoids and polyketides	Polyketide sugar unit biosynthesis<br>Terpenoid backbone biosynthesis<br>Biosynthesis of ansamycins<br>Biosynthesis of vancomycin group antibiotics
Nucleotide metabolism	Purine metabolism<br>Pyramidine metabolism
Xenobiotics biodegradation and metabolism	Chlorocyclohexane and chlorobenzene degradation<br>Fluorobenzoate degradation, Aminobenzoate degradation<br>Toluene degradation, Nitrotoluene degradation, Naphthalene degradation, Chloroalkane and chloroalkene degradation
